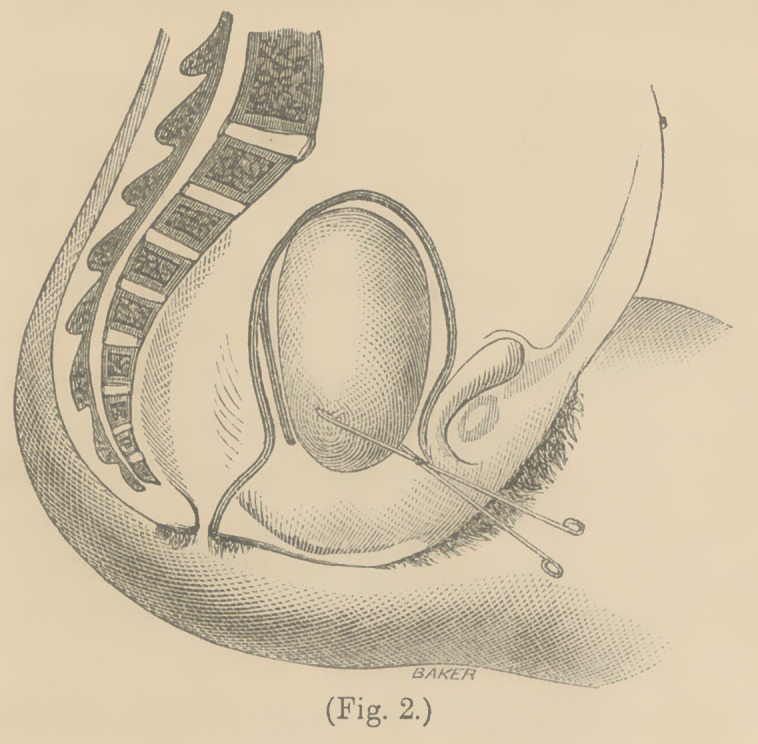# An Intra-Mural Fibrous Tumor Removed from the Anterior Wall of the Uterus

**Published:** 1869-01

**Authors:** William H. Byford

**Affiliations:** Professor of Obstetrics, etc., Chicago Medical College


### CHICAGO MEDICAL EXAMINER.

N. S. DAVIS, M.D., Editor.

VOL. X. JANUARY, 1869. NO. 1.

### Original Contributions.

### ARTICLE I.

### AN INTRA-MURAL FIBROUS TUMOR REMOVED FROM THE ANTERIOR WALL OF THE UTERUS.

By WILLIAM H. BYFORD, A.M., M.D., Professor of Obstetrics, etc., Chicago Medical College.

Mrs. McC., of Terre Haute, Indiana, is thirty-nine years of age; has been married twenty-one years; has four children, the first nineteen years old, the last eight, and had one miscarriage, twenty years since. Her health has been in every respect good until the last five years. Five years ago, she had severe neuralgic pain in her left side, extending to the hip and down the leg of the same side; from the history, most likely in the sciatic nerve and its branches; for about one month the limb was partially paralyzed. She has since then almost continually been the subject of indigestion, constipation, etc. Eighteen months since, she commenced having profuse discharges of blood from the vagina. These discharges had continued to increase up to the time when Dr. J. B. Buchtel was called to see her, on September 22d, 1868; when, according to the description given by Dr. Buchtel, she was anæmic to an extreme degree, with œdematous face and extremities, and effusion in the peritoneal cavity. She was almost constantly confined to her bed; had pain in the left iliac region, and down the left leg; was constipated, and vomited a part of her food, and was also much distressed with the digestion of what she retained. Her menses were regular, but profuse; besides this, she had profuse floodings between times, which were controlled only by the most active treatment; sometimes it was necessary to use the tampon. Between the hemorrhages she had profuse leucorrhœa. An examination made by Dr. Buchtel was followed by alarming hemorrhage. By this examination he discovered the presence of a large fibroid growth in the anterior wall of the uterus.

After a skilful and diligent course of treatment, consisting, for the most part, of tonics and alteratives, with good substantial diet, for about four weeks, Dr. Buchtel found his patient able to be brought to Chicago for advice and surgical treatment. October 24th, Mrs. McC.'s health very much improved, but still so feeble that she passed much of the time in bed. She expressed great fear of another paroxysm of hemorrhage. A careful examination confirmed Dr. Buchtel's diagnosis. There was a large, hard tumor imbedded in the anterior wall of the uterus. When the probe was passed into the cavity of that organ, and a catheter in the bladder, their lower extremities crossed each other, while the wide separation of their upper ends showed an intervening substance of about five inches. The lower end of the tumor was about on a level with the arch of the symphisis pubis, and had greatly developed and distended the anterior wall of the cervical part of the uterus, while the upper could be felt high up towards the umbilicus, a little more to the right than to the left of the linea alba.

(Fig. 1.)

The os uteri was soft and dilated, so that one finger would easily enter it. A good idea of the tumor *in situ* is given in Fig. 1. The bladder is crowded up to the symphisis, and the cavity of the uterus may be seen very greatly elongated behind the tumor.

I hoped to be able to destroy the vitality of the tumor by coring it after the method practised by Mr. Baker Brown, of London. With a view to an attempt of this kind, I placed the patient on the operating-table, on her left side, with her left arm behind her, so that she would lie well over on her breast, with the knees drawn up, the right bent the most, and drawn forward and over the left on the table. This is Sims's position for the operation for vesico-vaginal fistula. The introduction of a large-sized Sims speculum brought the lower end of the tumor full in view. After having anæsthetized the patient, in presence of the students of Chicago Medical College, at Mercy Hospital, assisted by Dr. Buchtel and some of the senior students, I commenced the operation.

An incision was first made in the most dependent part of the tumor, in the anterior lip of the uterus, which extended transversely from one side of the pelvis to the other, and must have been over three inches long; another, commencing in the centre of this, was extended up the posterior surface of the tumor, in the cavity of the uterus, as far as I could guide the scissors by the finger, with the hand partly introduced into the vagina. This last incision must have been more than three inches long also. The substance cut through was at least a quarter of an inch thick. The two incisions formed a ⊥-shaped opening into the cyst containing the tumor. The freedom with which I could separate the walls of the cyst from the tumor encouraged me to attempt the removal of the whole mass, instead of a part of it. For this purpose, I introduced my left hand into the vagina, and my fingers high up into the cyst; and after some exertion, had the satisfaction to break up the adhesions of the tumor to its envelope over much of its circumference, and well up towards the upper end.

I then seized the mass with a strong vulsel forceps, and made traction upon it in various directions, twisting, with a view to loosen it from its bed, and changing the bearing of the instrument in numerous ways, with apparently but little effect.

(Fig. 2.)

After much fatiguing effort, I passed the forceps up the posterior surface of the tumor, in the manner represented by Fig. 2, and made traction with great force, giving the instrument a swaying motion from side to side. Soon it became evident that the whole tumor was approaching the external orifice of the vagina. Thus I continued passing the forceps higher up from time to time, until, to my great delight, the whole mass engaged in the lower strait of the pelvis, through which it passed, after some resistance. The fingers were then passed up into the cavity of the cyst, in order to ascertain whether there was anything further to be removed. The uterus contracted very decidedly, and became firmer to the touch. I could not detect any other growth by the most careful examination. Not more than two ounces of blood was lost, and the woman exhibited no signs of exhaustion. No more than forty minutes elapsed from the time the patient was placed completely under the influence of ether until she was carried to her bed. No treatment but rest, and opiates enough to allay pain, was directed. Twenty drops of tincture of opium is all the medicines she required or took. There was no symptom requiring attention, but the patient seemed comfortable and cheerful from the time of the operation, and on the 10th of November she made the journey home.

December 1st I received a letter from Dr. Buchtel, saying that his patient was "quite well."

The tumor was fibroid, oval in shape, the small end down. It weighed twenty ounces avoirdupois, was five inches and a half long, four inches and three-quarters broad, and four and a quarter thick. It was so firm in structure, that the forcible efforts at removal did not mutilate it scarcely at all.

REMARKS.—The profession is anxiously collecting facts, and comparing the results, in the treatment of fibroid growths of the uterus, with a view to the formation of rules of practice. Heretofore, and at present, there is very little on the subject of management in reference to them definitively settled. Yet, when we look back for only a few years, we will find there has been progress enough to warrant the expectation that the future treatment of fibroids will be made better than the past.

It is with a view to assist in collecting material upon which to base rational methods of cure in the formidable conditions connected with them, that I record this case, and venture upon these remarks in connection with it. The successful enucleation of intra-mural fibroid tumors of the uterus is acknowledged to be the best mode of treatment, especially when their removal can be done at once; yet most instances are attended with many difficulties and dangers. The principal dangers are—1st, Serious damage to the uterus; 2d, Injury to other viscera, as the bladder, bowel, and peritoneal cavity; 3d, Hemorrhage; 4th, Subsequent inflammation; 5th, Toxæmia.

The most important difficulties—1st, The remote situation of the tumor; 2d, Contracted and undeveloped condition of the os and cervix uteri; 3d, Too great size of the growth to pass through the pelvis and cervix, after the latter is well developed.

I am aware that these are not all the dangers and difficulties met with; for every case will present its own peculiar difficulties; to be surmounted, at the time, by the ingenuity of the operator alone; but I think we have in those above mentioned such as are to be feared in the majority, if not the whole, of the cases operated upon. One of the most important, in fact indispensable, items preparatory to treatment, and which will enable us to avoid much of the dangers, and overcome many of the difficulties, is to definitely determine the relations of the tumor to the different parts of the uterus, the pelvic viscera, and the peritoneal sac.

A few very simple means of exploration are necessary, and, ordinarily, sufficient, for this purpose. The tumor should, in the first place, be pressed down as low in the pelvis as possible; the finger, in the second place, should be introduced as far up into the rectum as practicable; 3d, the probe passed up the cavity of the uterus; and 4th, a catheter introduced into the bladder. If the tumor is in the posterior wall of the organ, the finger and probe will be separated as widely as the thickness of the fibroid intervening. The finger may further determine something of the shape and consistence of the tumor, as also whether it occupies the median or lateral parts of the wall. If the growth is in the anterior wall, then the distance between the probe and catheter determines its size.

The hand pressed down from above in the abdominal cavity, while the probe is in the uterus, will enable us to judge pretty accurately the vertical dimensions. By this sort of exploration we definitely determine the position of the bladder and rectum, and may thus have the knowledge that will guide us clear of them, and point out the places where the peritoneal cavity approaches nearest the field of operation. With this knowledge, a careful operator would not be likely to inflict damage upon any of these organs.

The part of the uterus most in danger is that above the vaginal attachment, as it would seem that the segment ordinarily included in or pressed down into the vagina and developed by the tumor, will suffer almost any practicable mutilation, and recover from it without danger to life. It may be extensively excised and largely distended without serious hemorrhage, or other detriment resulting, as this and many other cases on record prove. If the vaginal portion is opened by incision or otherwise, so that the operator has free access to the cavity in which the tumor is imbedded, the uterine walls will bear great freedom of manipulation, on account of both their great elasticity and strength; for they are generally much hypertrophied.

The most distended portion of uterine tissue, in the case reported, was one-fourth of an inch thick; and it is reasonable to suppose that this part was not thicker than elsewhere. I think it may now be considered as true, that in very few cases is the danger from hemorrhage great; yet we should ever be watchful against the possibility of the contrary. The free use of iron, pulverized rhatany, cold, and, if need be, the tampon, are the resources available and effective. Nor does there seem to be just grounds for suspecting that the operation for enucleation and avulsion of these tumors will be followed by more violent or dangerous inflammation than amputations or other capital operations. The means of avoiding it are, to establish the best possible condition of health, by the administration of iron, nourishing diet, and cod-liver oil, before the operation, keep the bowels quiet and suppress pain by the moderate use of opium after it. Toxæmia will not be likely to occur if the cavity of the uterus, vagina, and sac of the tumor be kept clear of blood, or the debris and sloughs of the injured structures, by the free use of tepid water, and, if necessary, solutions of antiseptics.

When the tumor is high up, it may be impossible to reach it so as to attack it successfully; yet in many instances we may do much by pressing the tumor down from above. The undeveloped state of the cervix and closed condition of the mouth of the uterus may often be remedied in a short time by the admirable means we now possess for dilating them; but I believe the bruised and excited state of the tissues thus dilated is not as favorable for speedy recovery from damage as the condition left after simple incision, and should rather favor free division of the parts at the *time* of the operation.

It will be seen that much of the incision made in my case was outside the cavity of the cervix, and that the os was not dilated, except by dragging the tumor through it. The plan practised by Dr. Scott, in a case recently reported by him in the *California Medical Gazette*, of dividing the tumor when too large to be delivered otherwise, with the *ecrasseur* or scissors, until small enough to pass easily, is to be commended as a measure that will enable us to avoid the damage that might result from too great distention or pressure in forcing it through the external parts, and indispensable when too large to be delivered otherwise.

The only symptom that I think justifies an effort for the removal of intra-mural fibroids, where the operation is likely to be successful, is an exhausting or dangerous drain of blood; and even then the milder, though somewhat uncertain, measure recommended by Mr. Brown, of London, of incising the cervix, ought to be tried first, with the hope that nothing further will be necessary. Although these tumors of the uterus are very common, they comparatively seldom grow large enough to prove fatal by their size. Their presence alone, for the most part, is merely an inconvenience; but hemorrhage, when excessive, does great damage to the system—often directly, and oftener indirectly, bring about fatal results. We should not, therefore, operate because our patient has a tumor, but because the tumor is attended with damaging or dangerous hemorrhage.

## Figures and Tables

**Fig. 1. f1:**
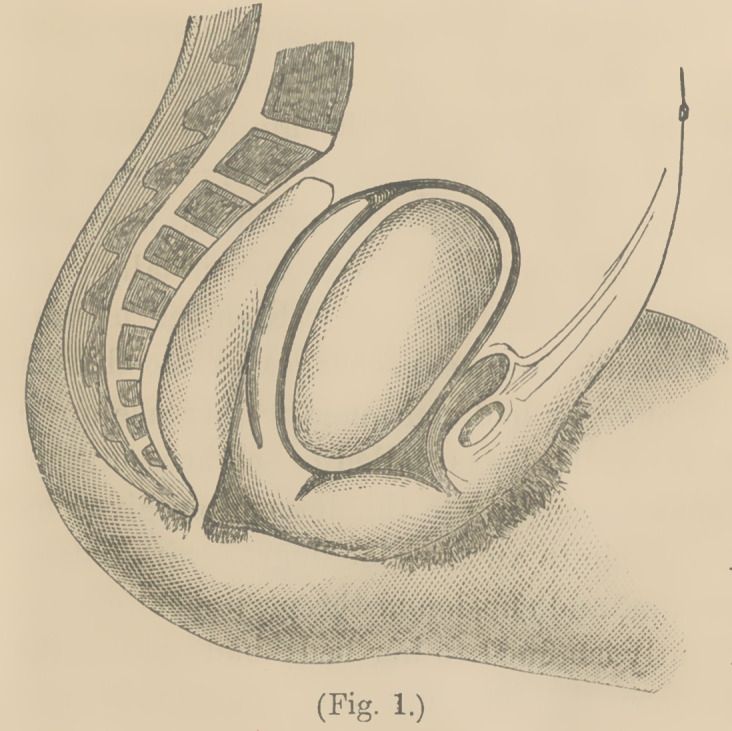


**Fig. 2. f2:**